# Supplementary material for: Microbial Community Shifts Associated With the Ongoing Stony Coral Tissue Loss Disease Outbreak on the Florida Reef Tract
Source: Front Microbiol. 2019 Sep 24;10:2244. doi: 10.3389/fmicb.2019.02244 (PMC6769089; doi:10.3389/fmicb.2019.02244)
Supplement: Supplementary file 1 [file Data_Sheet_1.PDF]

Table S1. Sample metadata for microbial communities collected from diseased and apparently healthy tissue associated with stony coral tissue loss disease. Condition DD indicates disease lesion, DH indicates apparently healthy tissue on diseased colonies (and is further parsed as near or far from the lesion), H indicates apparently healthy neighboring corals. Up to 3 samples were collected per coral colony, as indicated by the colony name. Raw reads (with adapters removed) are deposited in NCBI under Bioproject Accession # PRJNA521988.

Sample name	Site	Sample date	Coral	Colony	Condition	Near_Far	raw reads	reads used	NCBI Biosample Accession #
10FTL	Ft. Lauderdale	Dec-17	Montastraea cavernosa	mcav1	DD	lesion	163206	97735	SAMN10964620
11FTL	Ft. Lauderdale	Dec-17	Montastraea cavernosa	mcav1	DH	near	16564	11589	SAMN10964621
12FTL	Ft. Lauderdale	Dec-17	Montastraea cavernosa	mcav1	DH	far	32531	7695	SAMN10964622
13FTL	Ft. Lauderdale	Dec-17	Orbicella faveolata	ofav1	DH	far	77087	31860	SAMN10964623
14FTL	Ft. Lauderdale	Dec-17	Orbicella faveolata	ofav1	DH	near	52715	8627	SAMN10964624
15FTL	Ft. Lauderdale	Dec-17	Orbicella faveolata	ofav1	DD	lesion	59267	3288	SAMN10964625
1FTL	Ft. Lauderdale	Dec-17	Montastraea cavernosa	mcav2	DD	lesion	138377	26167	SAMN10964626
2FTL	Ft. Lauderdale	Dec-17	Montastraea cavernosa	mcav2	DH	near	126210	43396	SAMN10964627
3FTL	Ft. Lauderdale	Dec-17	Montastraea cavernosa	mcav2	DH	far	100050	19103	SAMN10964628
4FTL	Ft. Lauderdale	Dec-17	Montastraea cavernosa	mcav3	DD	lesion	9678	3513	SAMN10964629
5FTL	Ft. Lauderdale	Dec-17	Montastraea cavernosa	mcav3	DH	near	94544	15900	SAMN10964630
6FTL	Ft. Lauderdale	Dec-17	Montastraea cavernosa	mcav3	DH	far	37793	13040	SAMN10964631
7FTL	Ft. Lauderdale	Dec-17	Montastraea cavernosa	mcav4	DD	lesion	122318	77995	SAMN10964632
8FTL	Ft. Lauderdale	Dec-17	Montastraea cavernosa	mcav4	DH	near	151987	72875	SAMN10964633
9FTL	Ft. Lauderdale	Dec-17	Montastraea cavernosa	mcav4	DH	far	72044	7286	SAMN10964634
Dl10A	Long Key Bridge	Dec-17	Diploria labyrinthiformis	dl10	DD	lesion	175233	59052	SAMN10964635
Dl10B	Long Key Bridge	Dec-17	Diploria labyrinthiformis	dl10	DH	near	145483	49168	SAMN10964636
Dl10C	Long Key Bridge	Dec-17	Diploria labyrinthiformis	dl10	H	undiseased	162793	95389	SAMN10964637
Dl6A	Long Key Bridge	Dec-17	Diploria labyrinthiformis	dl6	DD	lesion	144530	119587	SAMN10964638
Dl6B	Long Key Bridge	Dec-17	Diploria labyrinthiformis	dl6	DH	near	107287	68671	SAMN10964639
Dl6C	Long Key Bridge	Dec-17	Diploria labyrinthiformis	dl6	DH	far	91447	44119	SAMN10964640
Dl7C	Long Key Bridge	Dec-17	Diploria labyrinthiformis	dl7	DH	far	111669	24228	SAMN10964641
Dl8A	Long Key Bridge	Dec-17	Diploria labyrinthiformis	dl8	DD	lesion	56113	20045	SAMN10964642
Dl8B	Long Key Bridge	Dec-17	Diploria labyrinthiformis	dl8	DH	near	85465	21961	SAMN10964643
Dl8C	Long Key Bridge	Dec-17	Diploria labyrinthiformis	dl8	DH	far	56643	2358	SAMN10964644
Dl9A	Long Key Bridge	Dec-17	Diploria labyrinthiformis	dl9	DD	lesion	159012	85597	SAMN10964645
Dl9B	Long Key Bridge	Dec-17	Diploria labyrinthiformis	dl9	DH	near	189498	40454	SAMN10964646
Dl9C	Long Key Bridge	Dec-17	Diploria labyrinthiformis	dl9	DH	far	244958	85801	SAMN10964647
Ds1A	Long Key Bridge	Dec-17	Dichocoenia stokesii	ds1	DD	lesion	159132	107824	SAMN10964648
Ds1B	Long Key Bridge	Dec-17	Dichocoenia stokesii	ds1	DH	near	72383	35677	SAMN10964649
Ds1C	Long Key Bridge	Dec-17	Dichocoenia stokesii	ds1	DH	far	100124	57394	SAMN10964650

Ds2A	Long Key Bridge	Dec-17	Dichocoenia stokesii	ds2	DD	lesion	71721	36451	SAMN10964651
Ds2B	Long Key Bridge	Dec-17	Dichocoenia stokesii	ds2	DH	near	64309	15308	SAMN10964652
Ds2C	Long Key Bridge	Dec-17	Dichocoenia stokesii	ds2	DH	far	51361	17476	SAMN10964653
Ds3A	Long Key Bridge	Dec-17	Dichocoenia stokesii	ds0	DD	lesion	164114	93611	SAMN10964654
Ds3B	Long Key Bridge	Dec-17	Dichocoenia stokesii	ds3	DD	lesion	137638	46421	SAMN10964655
Ds3C	Long Key Bridge	Dec-17	Dichocoenia stokesii	ds3	DH	far	156119	85671	SAMN10964656
Ds4A	Long Key Bridge	Dec-17	Dichocoenia stokesii	ds4	DD	lesion	111770	38470	SAMN10964657
Ds4B	Long Key Bridge	Dec-17	Dichocoenia stokesii	ds4	DH	near	102816	26344	SAMN10964658
Ds4C	Long Key Bridge	Dec-17	Dichocoenia stokesii	ds4	DH	far	122982	40126	SAMN10964659
PBa	Ft. Lauderdale	Jul-17	Montastraea cavernosa	a	H	undiseased	8605	3741	SAMN10964660
PBA1	Ft. Lauderdale	Jul-17	Montastraea cavernosa	A	DH	far	28274	14874	SAMN10964661
PBA2	Ft. Lauderdale	Jul-17	Montastraea cavernosa	A	DH	near	35187	9102	SAMN10964662
PBA3	Ft. Lauderdale	Jul-17	Montastraea cavernosa	A	DD	lesion	185193	109492	SAMN10964663
PBA4	Ft. Lauderdale	Jul-17	Montastraea cavernosa	A	DH	near	128398	44379	SAMN10964664
PBb	Ft. Lauderdale	Jul-17	Montastraea cavernosa	b	H	undiseased	6863	5478	SAMN10964665
PBB1	Ft. Lauderdale	Jul-17	Montastraea cavernosa	B	DH	far	68874	44928	SAMN10964666
PBB2	Ft. Lauderdale	Jul-17	Montastraea cavernosa	B	DH	near	79613	44691	SAMN10964667
PBB3	Ft. Lauderdale	Jul-17	Montastraea cavernosa	B	DD	lesion	338067	233271	SAMN10964668
PBc	Ft. Lauderdale	Jul-17	Montastraea cavernosa	c	H	undiseased	17609	10740	SAMN10964669
PBC1	Ft. Lauderdale	Jul-17	Montastraea cavernosa	C	DH	far	41706	27151	SAMN10964670
PBC2	Ft. Lauderdale	Jul-17	Montastraea cavernosa	C	DH	near	90670	37158	SAMN10964671
PBC3	Ft. Lauderdale	Jul-17	Montastraea cavernosa	C	DD	lesion	79129	67744	SAMN10964672
PBC4	Ft. Lauderdale	Jul-17	Montastraea cavernosa	c2	H	undiseased	3860	3116	SAMN10964673
PBd	Ft. Lauderdale	Jul-17	Montastraea cavernosa	d	H	undiseased	11947	9613	SAMN10964674
PBD1	Ft. Lauderdale	Jul-17	Montastraea cavernosa	D	DH	far	60338	37327	SAMN10964675
PBD2	Ft. Lauderdale	Jul-17	Montastraea cavernosa	D	DH	near	30105	9809	SAMN10964676
PBD3	Ft. Lauderdale	Jul-17	Montastraea cavernosa	D	DD	lesion	12906	10555	SAMN10964677
PBF1	Ft. Lauderdale	Jul-17	Montastraea cavernosa	f	H	undiseased	9843	5568	SAMN10964678
PBF2	Ft. Lauderdale	Jul-17	Montastraea cavernosa	F	DH	far	10623	8384	SAMN10964679
PBF3	Ft. Lauderdale	Jul-17	Montastraea cavernosa	F	DH	near	59953	26104	SAMN10964680
PBF4	Ft. Lauderdale	Jul-17	Montastraea cavernosa	F	DD	lesion	175522	118210	SAMN10964681
B1	extraction blank	n/a	n/a	n/a	n/a	n/a	1114	258	SAMN10964682
B2	extraction blank	n/a	n/a	n/a	n/a	n/a	97960	18348	SAMN10964683
B3	PCR blank	n/a	n/a	n/a	n/a	n/a	54582	11827	SAMN10964684
