# Supplementary material for: Microbial Community Shifts Associated With the Ongoing Stony Coral Tissue Loss Disease Outbreak on the Florida Reef Tract
Source: Front Microbiol. 2019 Sep 24;10:2244. doi: 10.3389/fmicb.2019.02244 (PMC6769089; doi:10.3389/fmicb.2019.02244)
Supplement: Supplementary file 2 [file Data_Sheet_2.PDF]

Table S2. Amplicon sequence variants detected in 3 control samples. Blank 1 (B1) and Blank 2 (B2) were no template controls from DNA extraction kit through sequencing. Blank 3 (B3) was a no template PCR control that was cleaned and sequenced. The total counts of these ASVs in all coral samples is also given for comparison.

Kingdom	Phylum	Class	Order	Family	Genus	blank total (n = 3)	B1	B2	B3	sample total (n = 62)	Amplicon Sequence Variant
Bacteria	Acidobacteria	Acidobacteriia	Solibacterales	Solibacteraceae_(Subgroup_3)	Bryobacter	157	0	0	157	0	TACGTAGGCAGCGAGCGTTGTTCGGAATTACTGGGCGTAAAGAGTGTGTAGGCGGTGCTCTAAGTTCGGTGTGAAATCTCCCGGCTTAACTGGGAGGGTGCGCCGGAAACTGGAGTGCTCGAGCGTGGGAGAGGAAAGCGGAATTCCTGGTGTAGCGGTGAAATGCGTAGATATCAGGAGGAACACCTGCGGTGTAGACGGCTTTCTGGACCATTGCTGACGCTGAGACACGAAAGCGTGGGTAGCAAACAGG
Bacteria	Acidobacteria	Acidobacteriia	Solibacterales	Solibacteraceae_(Subgroup_3)	Solibacteraceae_(Subgroup_3)	137	0	0	137	0	TACGTAGGCAGCGAGCGTTGTTCGGAATTACTGGGCGTAAAGAGTGTGTAGGCGGTGCTCTAAGTTCGGTGTGAAATCTCCCGGCTTAACTGGGAGGGTGCGCCGGAAACTGGAGTGCTCGAGCGTGGGAGAGGAAAGCGGAATTCCTGGTGTAGCGGTGAAATGCGTAGATATGTGGAAGAACACCCGTGGCGAAGGCGGCATCCTGGACCGGTATTGACGCTGATGCGCGAAAGCCAGGGGAGCAAACGGG
Bacteria	Actinobacteria	Actinobacteria	Corynebacteriales	Nocardiaceae	Gordonia	114	0	0	114	0	TACGTAGGGTGCGAGCGTTGTCCGGAATTACTGGGCGTAAAGAGCTCGTAGGCGGTTTGTCGCGTCGTCTGTGAAATTCTGCAGCTTAACTGCAGGCGTGCAGGCGATACGGGCAGACTTGAGTACTACAGGGGAGACTGGAATTCCTGGTGTAGCGGTGAAATGCGCAGATATCAGGAGGAACACCGGTGGCGAAGGCGGGTCTCTGGGTAGTAACTGACGCTGAGGAGCGAAAGCGTGGGTAGCGAACAGG
Bacteria	Actinobacteria	Actinobacteria	Corynebacteriales	Tsukamurellaceae	Tsukamurella	32	0	0	32	0	TACGTAGGGTGCGAGCGTTGTCCGGATTCACTGGGCGTAAAGAGCTCGTAGGCGGTTTGTCACGTCGTCTGTGAAAACCCGAGGCTTAACCTCGGGCCTGCAGGCGATACGGGCAGACTTGAGTACTGTAGGGGAGACTGGAATTCCTGGTGTAGCGGTGGAATGCGCAGATATCAGGAGGAACACCGGTGGCGAAGGCGGGTCTCTGGGCAGTAACTGACGCTGAGGAGCGAAAGCGTGGGTAGCGAACAGG
Bacteria	Actinobacteria	Actinobacteria	Micrococcales	Microbacteriaceae	Microbacteriaceae	261	0	261	0	0	TACGTAGGGTGCAAGCGTTGTCCGGAATTATTGGGCGTAAAGAGCTCGTAGGCGGTTTGTCACGTCTGCTGTGAAATTCCGAGGCTCAACCTCGGACTTGCAGTGGGTACGGGCAGGCTAGAGTGCGGTAGGGGAGATGGGAATTCCTGGTGTAGCGGTGGAATGCGTAGATATGCGGAGGAACACCGATGGCGAAGGCAATCCCCTGGGCCTGTACTGACGCTCATGCACGAAAGCGTGGGGAGCAAACAGG
Bacteria	Actinobacteria	Actinobacteria	Micrococcales	Microbacteriaceae	Parafrigoribacterium	352	0	0	352	0	TACGTAGGGTGCAAGCGTTATCCGGAATTATTGGGCGTAAAGAGCTCGTAGGCGGTTTGTCGCGTCTGCTGTGAAAACTGGAGGCTCAACCTCCAGCCTGCAGTGGGTACGGGCAGACTAGAGTGCGGTAGGGGAGATTGGAATTCCTGGTGTAGCGGTGGAATGCGCAGATATCAGGAGGAACACCAATGGCGAAGGCAGATCTCTGGGCCGTAACTGACGCTGAGGAGCGAAAGCATGGGGAGCGAACAGG
Bacteria	Actinobacteria	Actinobacteria	Micrococcales	Micrococcaceae	Renibacterium	270	15	0	255	3130	TACGTAGGGTGCAAGCGTTATCCGGAATTATTGGGCGTAAAGAGCTCGTAGGCGGTTTGTCGCGTCTGTCGTGAAAGTCCGGGGCTCAACTCCGGATCTGCGGTGGGTACGGGCAGACTAGAGTGATGTAGGGGAGACTGGAATTCCTGGTGTAGCGGTGGAATGCGCAGATATCAGGAGGAACACCGATGGCGAAGGCAGGTCTCTGGGCATTAACTGACGCTGAGGAGCGAAAGCATGGGGAGCGAACAGG
Bacteria	Actinobacteria	Actinobacteria	Micrococcales	Micrococcaceae	Renibacterium	35	0	35	0	0	TACGTAGGGTGCAAGCGTTATCCGGAATTATTGGGCGTAAAGAGCTCGTAGGCGGTTTGTCGCGTCTGTCGTGAAAGTCCGGGGCTCAACTCCGGATCTGCGGTGGGTACGGGCAGACTAGAGTGATGTAGGGGAGACTGGAATTCATGGTGTAGCGGTGGAATGCGCAGATATCAGGAGGAACACCGATGGCGAAGGCAGGTCTCTGGGCATTAACTGACGCTGAGGAGCGAAAGCATGGGGAGCGAACAGG
Bacteria	Actinobacteria	Actinobacteria	Pseudonocardiales	Pseudonocardiaceae	Actinomycetospora	96	0	0	96	0	TACGTAGGGTGCGAGCGTTGTCCGGAATTATTGGGCGTAAAGAGCTCGTAGGCGGTGTGTCACGTCGGCCGTGAAAACCTGGGGCTCAACTCTGGGCGTGCGGTCGATACGGGCATCACTTGAGTTCGGCAGGGGAGACTGGAATTCATGGTGTAGCGGTGAAATGCGCAGATATCAGGAGGAACACCGGTGGCGAAGGCGGGTCTCTGGGCCGATACTGACGCTGAGGAGCGAAAGCATGGGTAGCGAACAGG
Bacteria	Actinobacteria	Actinobacteria	Pseudonocardiales	Pseudonocardiaceae	Pseudonocardia	27	0	0	27	0	CACGTAGGGCGCGAGCGTTGTCCGGAATTATTGGGCGTAAAGAGCTCGTAGGCGGTGTGTCGCGTCGGCCGTGAAAACCTGCAGCTTAACTGTGGGCTTGCGGTCGATACGGGCATCACTGGAGTTCGGCAGGGGAGACTGGAATTCCTGGTGTAGCGGTGAAATGCGCAGATATCAGGAGGAACACCGGTGGCGAAGGCGGGTCTCTGGGCCGATACTGACGCTGAGGAGCGAAAGCGTGGGGAGCGAACAGG
Bacteria	Actinobacteria	Actinobacteria	Streptomycetales	Streptomycetaceae	Allostreptomyces	377	0	377	0	0	TACGTAGGGTGCGAGCGTTGTCCGGAATTATTGGACGTAAAGAGCTCGTAGGCGGCTTGTCGCGTCGGATGTGAAAGCCCGGGGCTTAACTCCGGGTCTGCATTCGATACGGGCAGGCTAGAGTTCGGTAGGGGAGATCGGAATTCCTGGTGTAGCGGTGAAATGCGCAGATATCAGGAGGAACACCGGTGGCGAAGGCAGCACTCTGGGACGTGACTGACGCTGAGACGCGAAAGCGTGGGGAGCAAACAGG
Bacteria	Actinobacteria	Actinobacteria	Streptomycetales	Streptomycetaceae	Streptomyces	150	0	0	150	0	TACGTAGGGCGCAAGCGTTGTCCGGAATTATTGGGCGTAAAGAGCTCGTAGGCGGCTTGTCACGTCGGACGTGAAAGCTTGGGGCTTAACTCCAAGTCTGCGTTCGATACGGGCTGGCTAGAGTGTGGTAGGGGAGATCGGAATTCCTGGTGTAGCGATGAAATGCGCAGATATCAGGAGGAACACCGGTGGCGAAGGCGGATCTCTGGGCCATTACTGACGCTGAGGAGCGAAAGCGTGGGGAGCGAACAGG
Bacteria	Actinobacteria	Actinobacteria	Streptomycetales	Streptomycetaceae	Streptomyces	130	0	0	130	0	TACGTAGGGCGCAAGCGTTGTCCGGAATTATTGGGCGTAAAGAGCTCGTAGGCGGCTTGTCACGTCGGACGTGAAAGCCCGGGGCTTAACCCCGGGTCTGCAGTCGATACGGGCAGGCTAGAGTTCGGTAGGGGAGATCGGAATTCCTGGTGTAGCGGTGAAATGCGCAGATATCAGGAGGAACACCGGTGGCGAAGGCGGATCTCTGGGCCGATACTGACGCTGAGGAGCGAAAGCGTGGGGAGCGAACAGG
Bacteria	Actinobacteria	Actinobacteria	Streptomycetales	Streptomycetaceae	Streptomycetaceae	1304	0	445	859	0	TACGTAGGGCGCAAGCGTTGTCCGGAATTATTGGGCGTAAAGAGCTCGTAGGCGGCTTGTCACGTCGGACGTGAAAGCTTGGGGCTTAACTCCAAGTCTGCGTTCGATACGGGCTGGCTAGAGTGTGGTAGGGGAGATCGGAATTCCTGGTGTAGCGGTGAAATGCGCAGATATCAGGAGGAACACCGGTGGCGAAGGCGGATCTCTGGGCCATTACTGACGCTGAGGAGCGAAAGCGTGGGGAGCGAACAGG
Bacteria	Actinobacteria	Actinobacteria	Streptomycetales	Streptomycetaceae	Streptomycetaceae	179	0	179	0	0	TACGTAGGGCGCAAGCGTTGTCCGGAATTATTGGGCGTAAAGAGCTCGTAGGCGGCTTGTCACGTCGGGTGTGAAAGCCCGGGGCTTAACCCCGGGTCTGCATTCGATACGGGCTGGCTAGAGTGTGGTAGGGGAGATCGGAATTCCTGGTGTAGCGGTGAAATGCGCAGATATCGGGAGGAACACCAGTGGCGAAGGCGGCTCACTGGACCACAACTGACGCTGAGGCGCGAAAGCTAGGGGAGCAAACAGG
Bacteria	Actinobacteria	Actinobacteria	Streptosporangiales	Streptosporangiaceae	Acrocarpospora	46	0	0	46	0	TACGTAGGGCGCAAGCGTTGTCCGGAATTATTGGGCGTAAAGAGCTCGTAGGCGGCCTGTTGCGTCTGCCGTGAAAGCCCGTGGCTTAACTGCGGGTCTGCGGTGGATACGGGCAGGCTAGAGGCTGGTAGGGGCAAGCGGAATTCCTGGTGTAGCGGTGAAATGCGCAGATATCAGGAGGAACACCGGTGGCGAAGGCGGCTTGCTGGGCCGGTTCTGACGCTGAGGAGCGAGAGCGTGGGGAGCGAACAGG
Bacteria	Actinobacteria	Rubrobacteria	Rubrobacterales	Rubrobacteriaceae	Rubrobacter	6	0	0	6	0	TACGTAGGGGGCGAGCGTTGTCCGGAATTATTGGGCGTAAAGGGCGCGTAGGCGGCCCGGTAAGTCTGCTGTGAAACCTTGGGGCTCAACCCCAAGCGTGCAGTGGATACTGTCGGGCTAGAGGATGGTAGAGGCCAGTGGAATTCCCGGTGTAGCGGTGAAATGCGCAGATATCGGGAGGAACACCAGTAGCGAAGGCGGCTGGCTGGGCCATTCCTGACGCTGAGGCGCGAAAGCTAGGGGAGCGAACAGG
Bacteria	Bacteria	Bacteria	Bacteria	Bacteria	Bacteria	368	0	368	0	19318	TACGTAGGGGGCAAACGTTGTTCGGAATCACTGGGCGTAAAGGGTATGTAGGTTGTTAAGTAAGTAAAAGGTGAAATCCCAAAGCTCAACTTTGGAATTGCTTTTTATACTGCTTAACTAGGGTCTCTTATAGAGGTAGTAGGAATTCCTAGTGGAGGAGTAAAATCTGTAGATATTAGGAGGAACACCAATGGCGAAGGCAAGCTACTGGGTAGAGACTGACATTGAGATACGAAAGCTAGGGGAGCAAATGGG
Bacteria	Bacteria	Bacteria	Bacteria	Bacteria	Bacteria	98	0	98	0	0	TACGAGTGCCCCGAGCGTTGTCCGGAATTACTGGGCGTAAAGGGTGCGTAGGGGGCGCTGTTAGTCCTGTTTTAAATCCTGGTGCTCAACATCAGGAGTGGGCGGGAAACGGCAGCGCTTAGAGGACGCGAGAGGTCTGCGGAACTCACGGTGTAGGGGTGAAATGCATAGATATTACACAGAACACCGATAGCGAAGGCAGCTCACTAGGCCTGGATTGACGCTCAGGGACGAAAGCGTGGGGATCAAACAGG
Bacteria	Bacteroidetes	Bacteroidia	Bacteroidales	Bacteroidaceae	Bacteroides	262	0	0	262	0	TACGGAGGATCCGAGCGTTATCCGGATTTATTGGGTTTAAAGGGAGCGTAGGTGGACAGTTAAGTCAGTTGTGAAAGTTTGCGGCTCAACCGTAAAATTGCAGTTGATACTGGCTGTCTTGAGTACAGTAGAGGTGGGCGGAATTCGTGGTGTAGCGGTGAAATGCTTAGATATCACGAAGAACTCCGATTGCGAAGGCAGCTCACTGGACTGCAACTGACACTGATGCTCGAAAGTGTGGGTATCAAACAGG
Bacteria	Bacteroidetes	Bacteroidia	Bacteroidales	Bacteroidaceae	Bacteroides	251	0	0	251	0	TACGGAGGATCCGAGCGTTATCCGGATTTATTGGGTTTAAAGGGAGCGTAGGTGGATTGTTAAGTCAGTTGTGAAAGTTTGCGGCTCAACCGTAAAATTGCAGTTGATACTGGATATCTTGAGTGCAGTTGAGGCAGGCGGAATTCGTGGTGTAGCGGTGAAATGCTTAGATATCACGAGGAACTCCGATTGCGAAGGCAGCTCACTGGACTGCAACTGACACTGATGCTCGAAAGTGTGGGTATCAAACAGG
Bacteria	Bacteroidetes	Bacteroidia	Bacteroidales	Bacteroidaceae	Bacteroides	57	0	0	57	0	TACGGAGGACCCGAGCGTTATCCGGATTTATTGGGTTTAAAGGGAGCGTAGGTGGACAGTTAAGTCAGTTGTGAAAGTTTGCGGCTCAACCGTAAAATTGCAGTTGATACTGGCTGTCTTGAGTACAGTAGAGGTGGGCGGAATTCGTGGTGTAGCGGTGAAATGCTTAGATATCACGAAGAACTCCGATTGCGAAGGCAGCTCACTGGACTGCAACTGACACTGATGCTCGAAAGTGTGGGTATCAAACAGG
Bacteria	Bacteroidetes	Bacteroidia	Bacteroidia	Bacteroidia	Bacteroidia	115	0	115	0	0	TACGAGTGCCCCGAGCGTTGTCCGGAATTACTGGGCGTAAAGGGTGCGTAGGGGGCGCTGTTAGTCCTGTTTTAAATCCTGGTGCTCAACATCAGGAGTGGGCGGGAAACGGCAGCGCTTAGAGGACGCGAGAGGTCTGCGGAACTCACGGTGTAGGGGTGAAATGCATAGATATTACACAGAACACCGATAGCGAAGGCAGCTCACTAGGCCTGGATTGACGCTCAGGGACGAAAGCGTGGGGAGCAAACAGG
Bacteria	Bacteroidetes	Bacteroidia	Chitinophagales	Chitinophagaceae	Chitinophaga	343	0	0	343	0	TACGGAGGGTGCAAGCGTTATCCGGATTCATTGGGTTTAAAGGGTGCGTAGTCGGATTTGTAAGTCCGTGGTGAAATCTCCAGGCTTAACCTGGAAACTGCCGTGGATACTATAAATCTTGAATGTTGTGGAGGTTAGCGGAATATGTCATGTAGCGGTGAAATGCATAGATATGACATAGAACACCAATTGCGAAGGCAGCTGGCTACACAAATATTGACGCTGAGGCACGAAAGCGTGGGGATCAAACAGG
Bacteria	Bacteroidetes	Bacteroidia	Chitinophagales	Chitinophagaceae	Chitinophaga	333	0	0	333	0	TACGGAGGGTGCAAGCGTTATCCGGATTCACTGGGTTTAAAGGGTGCGTAGGCGGATTTGTAAGTCCGTGGTGAAATCTCCAGGCTTAACCTGGAAACTGCCGTGGATACTATAAATCTTGAATGTTGTGGAGGTTAGCGGAATATGTCATGTAGCGGTGAAATGCATAGATATGACATAGAACACCAATTGCGAAGGCAGCTGGCTACACAAATATTGACGCTGAGGCACGAAAGCGTGGGGATCAAACAGG
Bacteria	Bacteroidetes	Bacteroidia	Cytophagales	Hymenobacteraceae	Hymenobacter	179	0	179	0	0	TACGGAGGGTGCGAGCGTTGTCCGGATTTATTGGGTTTAAAGGGTGCGTAGGCGGCTCATTAAGTCCGGGGTGAAAGCCCGTTGCTCAACAACGGAACTGCCCTGGATACTGGTGAGCTTGAGTACAGACGAGGTTGGCGGAATGGACCGAGTAGCGGTGAAATGCATAGATACGGTCCAGAACCCCGATTGCGAAGGCAGCTGACTAGGCTGTTACTGACGCTGAGGCACGACAGCGTGGGGAGCGAACAGG
Bacteria	Bacteroidetes	Bacteroidia	Cytophagales	Microscillaceae	Microscillaceae	42	0	42	0	0	TACGTAGGTGGCAAGCGTTGTCCGGATTTATTGGGTTTAAAGGGTGCGTAGGCGGTCTATTAAGTCAGTGGTGAAATACTCTAGCTCAACTAGAGGGGTGCCATTGATACTGATGGACTTGAGTACAGATGAGGTAGGCGGAATTGACGGTGTAGCGGTGAAATGCTTAGATATCGTCCAGAACACCGATAGCGAAGGCAGCTTACTAAGGAGTAACTGACGCTGAGGCACGAAAGTGTGGGGATCAAACAGG
Bacteria	Bacteroidetes	Bacteroidia	Flavobacteriales	Crocinitomicaceae	Fluviicola	29	29	0	0	0	TACGGAGGGTGCAAGCGTTGTCCGGATTTATTGGGTTTAAAGGGTGCGCAGGTGGTTTATTAAGTCAGTGGTGAAAGACGGTCGCTTAACGATAGAACTGCCATTGATACTGTAAGTCTTGAATTCGGTCGGAGTGGGCGGAATGTGTAGTGTAGCGGTGAAATGCATAGATATTACACAGAACACCGATAGCGAAGGCAGCTCACTAGGCCTGAATTGACGCTCAGGGACGAAAGCGTGGGGATCAAACAGG
Bacteria	Bacteroidetes	Bacteroidia	Flavobacteriales	Flavobacteriaceae	Flavobacteriaceae	16	16	0	0	0	TACGGAGGGTGCAAGCGTTATCCGGAATCATTGGGTTTAAAGGGTCCGTAGGCGGGCTATTAAGTCAGGGGTGAAAGTTTGTGGCTCAACCATAAAATTGCCCTTGATACTGGTAGTCTTGAGTTATTGTGAAGTGGTTGGAATATGTAGTGTAGCGGTGAAATGCATAGATATTACATAGAACACCGATTGCGAAGGCAGATCACTAACAATCGACTGACGCTGAGGTGCGAAAGTGTGGGGAGCAAACAGG
Bacteria	Bacteroidetes	Bacteroidia	Flavobacteriales	Flavobacteriaceae	Kordia	369	0	0	369	38	TACGGAGGATCCAAGCGTTATCCGGAATCATTGGGTTTAAAGGGTCCGCAGGCTGTTGTTTAAGTCAGAGGTGAAAGTTTGCAGCTCAACTGTAAAATTGCCTTTGATACTGGATGACTTGAGTTATAATGAAGTGGTTAGAATATGTAGTGTAGCGGTGAAATGCATAGATATTACATAGAATACCGATTGCGAAGGCAGATCACTAATTATAAACTGACGCTGAGGGACGAAAGCGTGGGGAGCGAACAGG

Bacteria	Bacteroidetes	Bacteroidia	Flavobacteriales	Flavobacteriaceae	Kordia	74	0	0	74	0	TACGGAGGACCCAAGCGTTATCCGGAATCATTGGGTTTAAAGGGTCCGCAGGCTGTTGTTTAAGTCAGAGGTGAAAGTTTGCAGCTCAACTGTAAAATTGCCTTTGATACTGGATGACTTGAGTTATAATGAAGTGGTTAGAATATGTAGTGTAGCGGTGAAATGCATAGATATTACATAGAATACCGATTGCGAAGGCAGATCACTAATTATAAACTGACGCTGAGGGACGAAAGCGTGGGGAGCGAACAGG
Bacteria	Cyanobacteria	Oxyphotobacteria	Synechococcales	Cyanobiaceae	Prochlorococcus_MIT9313	260	0	260	0	19107	TACGGGAGTGGCAAGCGTTATCCGGAATTATTGGGCGTAAAGCGTCCGCAGGCGGCTTTTCAAGTCTGCTGTTAAAGCGTGGAGCTTAACTCCATCATGGCAGTGGAAACTGAAAGGCTTGAGTATGGTAGGGGCAGAGGGAATTCCCGGTGTAGCGGTGAAATGCGTAGATATCGGGAAGAACACCAGTGGCGAAGGCGCTCTGCTGGGCCATTACTGACGCTCATGGACGAAAGCCAGGGGAGCGAAAGGG
Bacteria	Firmicutes	Bacilli	Bacillales	Bacillaceae	Bacillus	10	0	0	10	0	TACGTAGGTGGCAAGCGTTGTCCGGAATTATTGGGCGTAAAGCGCGCGCAGGCGGTTCCTTAAGTCTGATGTGAAAGCCCCCGGCTCAACCGGGGAGGGTCATTGGAAACTGGGGAACTTGAGTGCAGAAGAGGAGAGCGGAATTCCACGTGTAGCGGTGAAATGCGTAGAGATGTGGAGGAACACCAGTGGCGAAGGCGGCTCTCTGGTCTGTAACTGACGCTGAGGCGCGAAAGCGTGGGGAGCGAACAGG
Bacteria	Firmicutes	Bacilli	Bacillales	Family_X	Thermicanus	2	0	0	2	30	TACGTAGGGGGCGAGCGTTGTCCGGAATGATTGGGCGTAAAGCGCGCGCAGGCGGTCCTTTAAGTCTGATGTAAAAGCCCGCGGCTTAACCGCGGAAGGTCATTGGAAACTGGGGGACTTGAGGCTAGGAGAGGGAAGTGGAATTCCTGGTGTAGCGGTGAAATGCGTAGAGATCAGGAGGAATACCGATGGCGAAAGCAACTTCCTGGCTTAGAACTGACGCTGATGCGCGAAAGCGTGGGGAGCAAACAGG
Bacteria	Firmicutes	Bacilli	Bacillales	Paenibacillaceae	Brevibacillus	8	0	0	8	0	TACGTAGGTGGCAAGCGTTGTCCGGATTTATTGGGCGTAAAGCGCGCGCAGGCGGCTATGTAAGTCTGGTGTTAAAGCCCGGAGCTCAACTCCGGTTCGCATCGGAAACTGTGTAGCTTGAGTGCAGAAGAGGAAAGCGGTATTCCACGTGTAGCGGTGAAATGCGTAGAGATGTGGAGGAACACCAGTGGCGAAGGCGGCTTTCTGGTCTGTAACTGACGCTGAGGCGCGAAAGCGTGGGGAGCAAACAGG
Bacteria	Firmicutes	Bacilli	Lactobacillales	Streptococcaceae	Streptococcus	55	0	0	55	142	TACGTAGGTCCCGAGCGTTGTCCGGATTTATTGGGCGTAAAGCGAGCGCAGGCGGTTAGATAAGTCTGAAGTTAAAGGCTGTGGCTTAACCATAGTACGCTTTGGAAACTGTTTAACTTGAGTGCAAGAGGGGAGAGTGGAATTCCATGTGTAGCGGTGAAATGCGTAGATATATGGAGGAACACCGGTGGCGAAAGCGGCTCTCTGGCTTGTAACTGACGCTGAGGCTCGAAAGCGTGGGGAGCAAACAGG
Bacteria	Firmicutes	Clostridia	Clostridiales	Family_XII	Fusibacter	180	0	180	0	22159	TACGTAGGGGGCAAGCGTTATCCGGAATCACTGGGCGTAAAGGGAGTGTAGGCGGTCAAGCAAGTCAGAGGTGAAAGGCATCGGCTCAACCGGTGTAAGCCTTTGAAACTGTTTGACTTGAGTGCAGGAGAGGTAAGTGGAATTCCTAGTGTAGCGGTGAAATGCGTAGATATTAGGAGGAACACCAGTGGCGAAGGCGACTTACTGGACTGTAACTGACGCTGAGGCTCGAAAGCGTGGGGAGCGAACAGG
Bacteria	Firmicutes	Clostridia	Clostridiales	Ruminococcaceae	Ruminococcaceae_UCG-005	20	0	20	0	0	TACGTAGGTTGCAAGCGTTGTCCGGATTTACTGGGTGTAAAGGGCGTGTAGGCGGAGATGCAAGTTGGGAGTGAAATCCCGGGGCTCAACCCCGGAACTGCTCTCAAAACTGTATCCCTTGAGTATCGGAGAGGCAAGCGGAATTCCTAGTGTAGCGGTGAAATGCGTAGATATTAGGAGGAACACCAGTGGCGAAGGCGGCTTGCTGGACGACAACTGACGCTGAGGCGCGAAAGCGTGGGGAGCAAACAGG
Bacteria	Planctomycetes	Planctomycetacia	Pirellulales	Pirellulaceae	Pir4_lineage	141	0	141	0	0	TACGAACTGTGCGAACGTTATTCGGAATCACTGGGCTTAAAGGGTTTGTAGGCGGCCTTGTAAGTCAGGTGTGAAAGCCCTCGGCTCAACCGAGGAACAGCGCTTGATACTGCAAGGCTTGAGGGAGACAGGGGTAAGCGGAACTGATGGTGGAGCGGTGAAATGCGTTGATATCATCAGGAACACCGGTGGCGAAAGCGGCTTACTGGGTCTCTTCTGACGCTGAGGCGCGAAAGCCAGGGGAGCAAACGGG
Bacteria	Planctomycetes	Planctomycetacia	Pirellulales	Pirellulaceae	Pirellulaceae	23	23	0	0	0	TACAGAGGGGGCAAGCGTTGTTCGGAATTACTGGGCGTAAAGGGCGCGTAGGCGGCCCTCTAAGTCAGACGTGAAATCCCTCGGCTCAACCGAGGAACGGCGCTTGATACTGCAAGGCTTGAGGGAGACAGGGGTAAGCGGAACTGATGGTGGAGCGGTGAAATGCGTTGATATCATCAGGAACACCGGTGGCGAAAGCGGCTTACTGGGTCTCTTCTGACGCTGAGGAACGAAAGCTAGGGTAGCGAACGGG
Bacteria	Proteobacteria	Alphaproteobacteria	Acetobacterales	Acetobacteraceae	Rhodovarius	16	0	0	16	0	TACGAAGGGGGCTAGCGTTACTCGGAATGACTGGGCGTAAAGGGCGCGTAGGCTGTTTGGTAAGTTGGGCGTGAAATTCCTGGGCTTAACCTGGGGGCTGCGTCCAAGACTGCCTGACTTGAGGATGGAAGAGGCTCGTGGAATTCCCAGTGTAGAGGTGAAATTCGTAGATATTGGGAAGAACACCGGTGGCGAAGGCGGCGAGCTGGTCCATTACTGACGCTGAGGCGCGATAGCGTGGGGAGCAAACAGG
Bacteria	Proteobacteria	Alphaproteobacteria	Caulobacterales	Caulobacteraceae	Brevundimonas	14	0	14	0	0	TACGAAGGGGGCTAGCGTTGCTCGGAATTACTGGGCGTAAAGGGAGCGTAGGCGGACATTTAAGTCAGGGGTGAAATCCCAGAGCTCAACTCTGGAACTGCCTTTGATACTGGGTGTCTTGAGTGTGATAGAGGTATGTGGAACTCCGAGTGTAGAGGTGAAATTCGTAGATATTCGGAAGAACACCAGTGGCGAAGGCGACATACTGGATCATTACTGACGCTGAGGCTCGAAAGCGTGGGGAGCAAACAGG
Bacteria	Proteobacteria	Alphaproteobacteria	Caulobacterales	Caulobacteraceae	Caulobacter	12	0	12	0	0	TACGAAGGGGGCTAGCGTTGCTCGGAATTACTGGTCGTAAAGGGAGCGTAGGCGGACTGTTTAGTCAGAGGTGAAAGCCCAGGGCTCAACCTTGGAATTGCCTTTGATACTGGCAGTCTTGAGTACGGAAGAGGTATGTGGAACTCCGAGTGTAGAGGTGAAATTCGTAGATATTCGGAAGAACACCAGTGGCGAAGGCGACATACTGGTCCGTTACTGACGCTGAGGCTCGAAAGCTTGGGGAGCAAACAGG
Bacteria	Proteobacteria	Alphaproteobacteria	Rhizobiales	Amb-16S-1323	Amb-16S-1323	76	0	0	76	0	TACGAAGGGGGCTAGCGTTGCTCGGAATCACTGGGCGTAAAGCGCACGTAGGTGGGTCATTTAGTCAGGGGTGAAATCCTGGAGCTCAACTCCAGAACTGCCTTTGATACTGATGATCTTCGAGTCCGGGAGAGGTGGGTGGAACTGCGAGTGTAGAGGTGAAATTCGTAGATATTCGCAAGAACACCAGTGGCGAAGGCGGCTCACTGGCCCGGTACTGACGCTGAGGTGCGAAAGCGTGGGGAGCAAACAGG
Bacteria	Proteobacteria	Alphaproteobacteria	Rhizobiales	Devosiaceae	Devosia	14	0	0	14	0	TACGAAGGGGGCTAGCGTTGTTCGGATTTACTGGGCGTAAAGCGCACGTAGGCGGTTTGTTAAGTCAGGGGTGAAATCCCGGAGCTCAACTCCGGAACTGCCTTTGATACTGGCAAGCTAGAGTCCGGAAGAGGTAAGTGGAACTCCTAGTGTAGAGGTGGAATTCGTAGATATTAGGAAGAACACCAGTGGCGAAGGCGGCTTACTGGTCCGGAACTGACGCTGAGGTGCGAAAGCGTGGGGAGCAAACAGG
Bacteria	Proteobacteria	Alphaproteobacteria	Rhizobiales	Methyloligellaceae	Methyloligellaceae	184	0	0	184	0	TACGGAGGGGACTAGCGTTGTTCGGAATTACTGGGCGTAAAGCGCACGTAGGCGGATTTGTAAGTCAGGGGTGAAATCCCGAGGCTCAACCTCGGAACTGCCTTTGATACTGCAAATCTCGAGTCCGGAAGAGGTGAGTGGAATTCCTAGTGTAGAGGTGAAATTCGTAGATATTAGGAAGAACACCAGTGGCGAAGGCGGCTCACTGGTCCGGTACTGACGCTGAGGTGCGAAAGCGTGGGGAGCAAACAGG
Bacteria	Proteobacteria	Alphaproteobacteria	Rhizobiales	Rhizobiaceae	Candidatus_Liberibacter	53	0	53	0	280	TACGAAGGGGGCGAGCGTTGTTCGGAATAACTGGGCGTAAAGGGCGCGTAGGCGGGCGATTAAGTTAGAGGTGAAATCCCAGGGCTCAACCTTGGAACTGCCTTTAATACTGGTTGTCTAGAGTTTAGGAGAGGTGAGTGGAATTCCGAGTGTAGAGGTGAAATTCGTAGATATTCGGAGGAACACCGGTGGCGAAGGCGGCTCACTGGCCTGATACTGACGCTGAGGCGCGAAAGCGTGGGGAGCAAACAGG
Bacteria	Proteobacteria	Alphaproteobacteria	Rhizobiales	Rhizobiales	Rhizobiales	305	0	0	305	0	TACGGAGGGGACTAGCGTTGTTCGGAATTACTGGGCGTAAAGCGCACGTAGGCGGATTTGTAAGTCAGGGGTGAAATCCCGAGGCTCAACCTCGGAACTGCCTTTGATACTGCAAGTCTCGAGTCCGGAAGAGGTGAGTGGAATTCCTAGTGTAGAGGTGAAATTCGTAGATATTAGGAAGAACACCAGTGGCGAAGGCGGCTCACTGGTCCGGTACTGACGCTGAGGTGCGAAAGCGTGGGGAGCAAACAGG
Bacteria	Proteobacteria	Alphaproteobacteria	Rhizobiales	Rhizobiales	Rhizobiales	225	0	0	225	0	TACGAAGGGGGCTAGCGTTGTTCGGATTTACTGGGCGTAAAGCGCGTGTAGGCGGGTCGTTAAGTTGGGGGTGAAATCCCGAGGCTCAACCTCGGAACTGCCTTTGATACTGCAAATCTCGAGTCCGATAGAGGTGAGTGGAACTGCGAGTGTAGAGGTGAAATTCGTAGATATTCGCAAGAACACCAGTGGCGAAGGCGGCTCACTGGTCCGGTACTGACGCTGAGGTGCGAAAGCGTGGGGAGCAAACAGG
Bacteria	Proteobacteria	Alphaproteobacteria	Rhizobiales	Xanthobacteraceae	Bradyrhizobium	446	0	0	446	0	TACGAAGGGGGCTAGCGTTGCTCGGAATCACTGGGCGTAAAGGGTGCGTAGGCGGGTCTTTAAGTCAGGGGTGAAATCCTGGAGCTCAACTCCAGAACTGCCTTTGATACTGAAGATCTTGAGTTCGGGAGAGGTGAGTGGAACTGCGAGTGTAGAGGTGAAATTCGTAGATATTCGCAAGAACACCAGTGGCGAAGGCGGCTCACTGGCCCGATACTGACGCTGAGGTGCGAAAGCGTGGGGAGCAAACAGG
Bacteria	Proteobacteria	Alphaproteobacteria	Rhizobiales	Xanthobacteraceae	Xanthobacteraceae	415	0	415	0	0	TACGAAGGGGGCTAGCGTTGTTCGGAATCACTGGGCGTAAAGGGTGCGTAGGCGGGTCTTTAAGTCAGGGGTGAAATCCTGGAGCTCAACTCCAGAACTGCCTTTGATACTGGCGATCTTGAGTTCGGGAGAGGTGAGTGGAACTGCGAGTGTAGAGGTGAAATTCGTAGATATTCGCAAGAACACCAGTGGCGAAGGCGGCTACCTGGCCCAATACTGACACTCAGGTGCGACAGCGTGGGGAGCAAACAGG
Bacteria	Proteobacteria	Alphaproteobacteria	Rhizobiales	Xanthobacteraceae	Xanthobacteraceae	161	0	0	161	0	TACGAAGGGTGCAAGCGTTACTCGGAATCACTGGGCGTAAAGCGCACGTAGGCGGATCGTTAAGTCAGGGGTGAAATCCTGGAGCTCAACTCCAGAACTGCCTTTGATACTGGCGACCTTGAGTTCGGGAGAGGTGAGTGGAACTGCGAGTGTAGAGGTGAAATTCGTAGATATTCGCAAGAACACCAGTGGCGAAGGCGGCTCACTGGCCCGATACTGACGCTGAGGTGCGAAAGCGTGGGGAGCAAACAGG
Bacteria	Proteobacteria	Alphaproteobacteria	Rhizobiales	Xanthobacteraceae	Xanthobacteraceae	128	0	0	128	0	TACGAAGGGGGCTAGCGTTGCTCGGAATCACTGGGCGTAAAGCGCACGTAGGCGGATCGTTAAGTCAGGGGTGAAATCCTGGAGCTCAACTCCAGAACTGCCTTTGATACTGGCGACCTTGAGTTCGGGAGAGGTGAGTGGAACTGCGAGTGTAGAGGTGAAATTCGTAGATATTCGCAAGAACACCAGTGGCGAAGGCGGCTCACTGGCCCGATACTGACGCTGAGGTGCGAAAGCGTGGGGAGCAAACAGG
Bacteria	Proteobacteria	Alphaproteobacteria	Rhizobiales	Xanthobacteraceae	Xanthobacteraceae	41	0	0	41	0	TACGAAGGGCGCAAGCGTTACTCGGAATCACTGGGCGTAAAGCGCACGTAGGCGGATCGTTAAGTCAGGGGTGAAATCCTGGAGCTCAACTCCAGAACTGCCTTTGATACTGGCGACCTTGAGTTCGGGAGAGGTGAGTGGAACTGCGAGTGTAGAGGTGAAATTCGTAGATATTCGCAAGAACACCAGTGGCGAAGGCGGCTCACTGGCCCGATACTGACGCTGAGGTGCGAAAGCGTGGGGAGCAAACAGG
Bacteria	Proteobacteria	Alphaproteobacteria	Rhodobacterales	Rhodobacteraceae	Rhodobacteraceae	316	0	316	0	706	TACGGAGGGGGTTAGCGTTGTTCGGAATTACTGGGCGTAAAGCGCGCGTAGGCGGATTGGAAAGTTAGAGGTGAAATCCCGGGGCTCAACCCCGGAACTGCCTTTAAAACTCCCAGTCTTGAGTTCGAGAGAGGTGAGTGGAACTCCGAGTGTAGAGGTGAAATTCGTAGATATTCGGAAGAACACCAGTGGCGAAGGCGGCTCACTGGCTCGATACTGACGCTGAGGTGCGAAAGTGTGGGGAGCAAACAGG
Bacteria	Proteobacteria	Alphaproteobacteria	Rhodobacterales	Rhodobacteraceae	Rhodobacteraceae	300	0	0	300	558	TACGGAGGGGGTTAGCGTTGTTCGGAATTACTGGGCGTAAAGCGCGCGTAGGCGGACCAGAAAGTTGGGGGTGAAATCCCGGGGCTCAACCCCGGAACTGCCTCCAAAACTCCTGGTCTAGAGTTCGAGAGAGGTGAGTGGAATTCCGAGTGTAGAGGTGAAATTCGTAGATATTCGGAGGAACACCAGTGGCGAAGGCGGCTCACTGGCTCGATACTGACGCTGAGGTGCGAAAGTGTGGGGAGCAAACAGG
Bacteria	Proteobacteria	Alphaproteobacteria	Rhodobacterales	Rhodobacteraceae	Rhodobacteraceae	152	0	0	152	0	TACGGAGGGGGTTAGCGTTGTTCGGAATTACTGGGCGTAAAGCGCGCGTAGGCGGATTGGAAAGTTGGGGGTGAAATCCCGGGGCTCAACCCCGGAACTGCCTCCAAAACTCCCAGTCTCGAGGATGAGAGAGGCAAGTGGAATACCGAGTGTAGAGGTGAAATTCGTAGATATTCGGTGGAACACCCGTGGCGAAGGCGGCTTGCTGGCTCATTTCTGACGCTGAGGTGCGAAAGCGTGGGGAGCAAACAGG
Bacteria	Proteobacteria	Alphaproteobacteria	Rhodobacterales	Rhodobacteraceae	Rhodobacteraceae	141	0	0	141	0	TACGGAGGGGGCTAGCGTTGTTCGGAATTACTGGGCGTAAAGCGCGCGTAGGCGGACTGGAAAGTTGGGGGTGAAATCCCAGGGCTCAACCCTGGAACTGCCTTCAAAACTCCCAGTCTCGAGGATGAGAGAGGCAAGTGGAATACCGAGTGTAGAGGTGAAATTCGTAGATATTCGGTGGAACACCCGTGGCGAAGGCGGCTTGCTGGCTCATTTCTGACGCTGAGGTGCGAAAGCGTGGGGAGCAAACAGG
Bacteria	Proteobacteria	Alphaproteobacteria	Rickettsiales	Midichloriaceae	MD3-55	1242	32	1210	0	0	TACGAAGGGGGCAAGCGTTACTCGGAATTATTGGGCGTAAAGCGTGCGTAGGCGGTTTTATAAGTTGAAAGTGAAAGCCTTTGGCTCAACCAAAGAATTGCTTACAAAACTGTAAAACTAGAGTATTAGAGAAGATAGAAGAATTCCTGATGTAGGGGTGAAATCCGTAGATATCAGGAGGAATATCGAAGGCGAAAGCATCTGTCTGGCTAAATACTGACGCTGTTGCACGAAAGCGTGGGGAGCAAACAGG
Bacteria	Proteobacteria	Alphaproteobacteria	Rickettsiales	Midichloriaceae	MD3-55	405	0	405	0	0	TACGAAGGGAGCAAGCGTTACTCGGAATTATTGGGCGTAAAGCGTGCGTAGGCGGTTTTATAAGTTGAAAGTGAAAGCCTTTGGCTCAACCAAAGAATTGCTTACAAAACTGTAAAACTAGAGTATTAGAGAAGATAGAAGAATTCCTGATGTAGGGGTGAAATCCGTAGATATCAGGAGGAATATCGAAGGCGAAAGCATCTGTCTGGCTAAATACTGACGCTGTTGCACGAAAGCGTGGGGAGCAAACAGG
Bacteria	Proteobacteria	Alphaproteobacteria	Rickettsiales	Midichloriaceae	MD3-55	195	0	195	0	4441	TACGAAGGGGGCAAGCGTTACTCGGAATTATTGGGCGTAAAGCGTGCGTAGGCGGTTTTATAAGTTGAAAGTGAAAGCCTTTGGCTCAACCAAAGAATTGCTTACAAAACTGTAAAACTAGAGTATTAGAGAGGATAGAAGAATTCCTGATGTAGGGGTGAAATCCGTAGATATCAGGAGGAATATCGAAGGCGAAAGCATCTGTCTGGCTAAATACTGACGCTGTTGCACGAAAGCGTGGGGAGCAAACAGG

Bacteria	Proteobacteria	Alphaproteobacteria	Rickettsiales	Midichloriaceae	MD3-55	78	0	78	0	0	TACGAAGGGGGCAAGCGTTACTCTGAATTATTGGGCGTAAAGCGTGCGTAGGCGGTTTTATAAGTTGAAAGTGAAAGCCTTTGGCTCAACCAAAGAATTGCTTACAAAACTGTAAAACTAGAGTATTAGAGAAGATAGAAGAATTCCTGATGTAGGGGTGAAATCCGTAGATATCAGGAGGAATATCGAAGGCGAAAGCATCTGTCTGGCTAAATACTGACGCTGTTGCACGAAAGCGTGGGGAGCAAACAGG
Bacteria	Proteobacteria	Alphaproteobacteria	Sphingomonadales	Sphingomonadaceae	Altererythrobacter	24	0	0	24	0	TACGGAGGGAGCTAGCGTTGTTCGGAATTACTGGGCGTAAAGCGCGCGTAGGCGGCTATTCAAGTCAGAGGTGAAATCCCGGGGCTCAACCCCGGAACTGCCTTTGAAACTGGATGGCTAGAATCCTGGAGAGGCGAGTGGAATTCCGAGTGTAGAGGTGAAATTCGTAGATATTCGGAAGAACACCAGTGGCGAAGGCGACTCGCTGGACAGGTATTGACGCTGAGGTGCGAAAGCGTGGGGAGCAAACAGG
Bacteria	Proteobacteria	Deltaproteobacteria	Myxococcales	bacteriap25	bacteriap25	6	0	0	6	0	TACAGAGGGCGCTAGCGTTGTTCGGAATCATTGGGCGTAAAGGGCGTGTAGGCGGTTTGCTAAGTCATGTGTGAAATCCCTCGGCTCAACCGGGGAACGACGCCTGAAACTGGCAAGCTAGAGTACCAAAGAGGGGGGTGGAATTCCCGGTGTAGCGGTGAAATGCGTAGATATCGGGAGGAACACCTGTGGCGAAGGCGGCCCCCTGGTTGGATACTGACGCTGAGACGCGAAAGCGTGGGGAGCAAACAGG
Bacteria	Proteobacteria	Deltaproteobacteria	Myxococcales	Myxococcales	Myxococcales	339	0	0	339	0	TACGAAGGGGGCGAGCGTTGTTCGGATTTACTGGGCGTAAAGGGCGCGCAGGCGGCGGACCAAGTCAGGTGTGAAAGCCCCAGGCTTAACCTGGGAAGTGCACTCGAAACTGCAGGGCTTGAGTATGGAAGAGGGTCTCGGAATTCCCGGTGTAGAGGTGAAATTCGTAGATATCGGGAGGAACACCAGTGGCGAAGGCGGAGACCTGGGCCAATACTGACGCTGAGGTGCGAAAGCGTGGGGAGCAAACAGG
Bacteria	Proteobacteria	Deltaproteobacteria	Myxococcales	P3OB-42	P3OB-42	21	0	21	0	0	TACAGAGGGCGCAAACGTTGCTCGGAATGACTGGGCGTAAAGCGCGTAGGCGGTGGGTTAAGTCGAATGTGAAAGCCCATGGCTCAACCATGGAAGCGCATTCGAAACTGGCTCACTGGAGTCCCGGAGAGGGTGGTGGAATTCCTAGTGTAGAGGTGAAATTCGTAGAGATTAGGAGGAACACCGGTGGCGAAGGCGACCATCTGGACGGGTACTGACGCTGAGGCGCGAAAGCGTGGGTAGCGAACAGG
Bacteria	Proteobacteria	Gammaproteobacteria	Alteromonadales	Shewanellaceae	Shewanella	264	0	264	0	90723	TACGGAGGGTGCGAGCGTTAATCGGAATTACTGGGCGTAAAGCGTGCGCAGGCGGTTTGTTAAGCGAGATGTGAAAGCCCCGGGCTCAACCTGGGAACCGCATTTCGAACTGGCAAACTAGAGTCTTGTAGAGGGGGGTAGAATTCCAGGTGTAGCGGTGAAATGCGTAGAGATCTGGAGGAATACCGGTGGCGAAGGCGGCCCCCTGGACAAAGACTGACGCTCAGGCACGAAAGCGTGGGGAGCAAACAGG
Bacteria	Proteobacteria	Gammaproteobacteria	Alteromonadales	Shewanellaceae	Shewanella	110	0	110	0	0	TACGGAGGGTGCGAGCGTTAATCGGAATTACTGGGCGTAAAGCGTGCGCAGGCGGTTTGTTAAGCGAGATGTGAAAGCCCCGGGCTCAACCTGGGAACCGCATTTCGAACTGGCAAACTAGAGTATTGTAGAGGGGGGTAGAATTCCAGGTGTAGCGGTGAAATGCGTAGAGATCTGGAGGAATACCGGTGGCGAAGGCGGCCCCCTGGACAAAGACTGACGCTCAGGCACGAAAGCGTGGGGAGCAAACAGG
Bacteria	Proteobacteria	Gammaproteobacteria	Betaproteobacteriales	Burkholderiaceae	Acidovorax	93	0	0	93	21	TACGTAGGGTGCAAGCGTTAATCGGAATTACTGGGCGTAAAGCGTGCGCAGGCGGTTATATAAGACAGATGTGAAATCCCCGGGCTCAACCTGGGAACTGCATTTGTGACTGTATAGCTAGAGTACGGCAGAGGGGGATGGAATTCCGCGTGTAGCAGTGAAATGCGTAGATATGCGGAGGAACACCGATGGCGAAGGCAATCCCCTGGGCCTGTACTGACGCTCATGCACGAAAGCGTGGGGAGCAAACAGG
Bacteria	Proteobacteria	Gammaproteobacteria	Betaproteobacteriales	Burkholderiaceae	Burkholderia-Caballeronia-Paraburkholderia	295	0	0	295	0	TACGTAGGGTGCGAGCGTTAATCGGAATTACTGGGCGTAAAGCGTGCGCAGGCGGTGATGTAAGACCGATGTGAAATCCCCGGGCTTAACCTGGGAACTGCATTGGTGACTGCATCGCTGGAGTATGGCAGAGGGGGGTAGAATTCCACGTGTAGCAGTGAAATGCGTAGAGATGTGGAGGAATACCGATGGCGAAGGCAGCCCCCTGGGCCAATACTGACGCTCATGCACGAAAGCGTGGGGAGCAAACAGG
Bacteria	Proteobacteria	Gammaproteobacteria	Betaproteobacteriales	Burkholderiaceae	Burkholderia-Caballeronia-Paraburkholderia	245	0	0	245	0	TACGTAGGGTGCAAGCGTTAATCGGAATTACTGGGCGTAAAGCGTGCGCAGGTGGTTATGTAAGACAGATGTGAAATCCCCGGGCTTAACCTGGGAACTGCATTGGTGACTGGCGGGCTGGAGTATGGCAGAGGGGGGTAGAATTCCACGTGTAGCAGTGAAATGCGTAGAGATGTGGAGGAATACCGATGGCGAAGGCAGCCCCCTGGGCCAATACTGACGCTCATGCACGAAAGCGTGGGGAGCAAACAGG
Bacteria	Proteobacteria	Gammaproteobacteria	Betaproteobacteriales	Burkholderiaceae	Burkholderiaceae	263	0	0	263	0	TACGTAGGGTGCAAGCGTTAATCGGAATTACTGGGCGTAAAGCGTGCGCAGGCGGCTTTGCAAGACAGATGTGAAATCCCCGGGCTTAACCTGGGAACTGCATTTGTGACTGCATGGCTGGAGTGTGGCAGAGGGGGATGGAATTCCGCGTGTAGCAGTGAAATGCGTAGATATCTGGAGGAACACCGGTGGTGAAGGCTGCGTCCTGGACCAATACTGACACTGAGGAGCGAAAGCCAGGTGAGCAAACGGG
Bacteria	Proteobacteria	Gammaproteobacteria	Betaproteobacteriales	Burkholderiaceae	Cupriavidus	439	0	0	439	0	TACGTAGGGTGCGAGCGTTAATCGGAATTACTGGGCGTAAAGCGTGCGCAGGCGGTTTTGTAAGACAGGCGTGAAATCCCCGAGCTCAACTTGGGAATTGCGCTTGTGACTGCAAGGCTAGAGTATGTCAGAGGGGGGTAGAATTCCACGTGTAGCAGTGAAATGCGTAGAGATGTGGAGGAATACCGATGGCGAAGGCAGCCCCCTGGGACGTCACTGACGCTCATGCACGAAAGCGTGGGGAGCAAACAGG
Bacteria	Proteobacteria	Gammaproteobacteria	Betaproteobacteriales	Burkholderiaceae	Cupriavidus	44	0	0	44	32	TACGTAGGGTGCGAGCGTTAATCGGAATTACTGGGCGTAAAGCGTGCGCAGGCGGTTTTGTAAGACAGGCGTGAAATCCCCGGGCTTAACCTGGGAATTGCGCTTGTGACTGCAAGGCTAGAGTGCGTCAGAGGGGGGTAGAATTCCACGTGTAGCAGTGAAATGCGTAGAGATGTGGAGGAATACCGATGGCGAAGGCAGCCCCCTGGGACGTGACTGACGCTCATGCACGAAAGCGTGGGGAGCAAACAGG
Bacteria	Proteobacteria	Gammaproteobacteria	Betaproteobacteriales	Burkholderiaceae	Polynucleobacter	46	46	0	0	0	TACGTAGGGTGCAAGCGTTAATCGGAATTACTGGGCGTAAAGCGTGCGCAGGCGGTTATGTAAGACAGGCGTGAAATCCCCGGGCTTAACCTGGGAATTGCGCTTGTGACTGCATAGCTAGAGTATGTCAGAGGGGGGTAGAATTCCACGTGTAGCAGTGAAATGCGTAGAGATGTGGAGGAATACCAATGGCGAAGGCAGCCCCCTGGGATAATACTGACGCTCATGCACGAAAGCGTGGGGAGCAAACAGG
Bacteria	Proteobacteria	Gammaproteobacteria	Betaproteobacteriales	Burkholderiaceae	Ralstonia	20	0	0	20	4	TACGTAGGGTCCAAGCGTTAATCGGAATTACTGGGCGTAAAGCGTGCGCAGGCGGTTGTGCAAGACCGATGTGAAATCCCCGGGCTTAACCTGGGAATTGCATTGGTGACTGCACGGCTAGAGTGTGTCAGAGGGAGGTAGAATTCCACGTGTAGCAGTGAAATGCGTAGAGATGTGGAGGAATACCGATGGCGAAGGCAGCCTCCTGGGATAACACTGACGCTCATGCACGAAAGCGTGGGGAGCAAACAGG
Bacteria	Proteobacteria	Gammaproteobacteria	Betaproteobacteriales	Burkholderiaceae	Tepidimonas	11	0	11	0	0	TACGTAGGGTGCGAGCGTTAATCGGAATTACTGGGCGTAAAGCGTGCGCAGGCGGTTGTGCAAGACAGGCGTGAAATCCCCGGGCTTAACCTGGGAATGGCGCTTGTGACTGCACGGCTGGAGTGCGGCAGAGGGGGATGGAATTCCGCGTGTAGCAGTGAAATGCGTAGATATGCGGAGGAACACCGATGGCGAAGGCAATCCCCTGGGCCTGCACTGACGCTCATGCACGAAAGCGTGGGGAGCAAACAGG
Bacteria	Proteobacteria	Gammaproteobacteria	Betaproteobacteriales	Nitrosomonadaceae	IS-44	253	0	0	253	0	TACGTAGGGTGCAAGCGTTAATCGGAATTACTGGGCGTAAAGCGTGCGCAGGCGGCTTTGCAAGACAGATGTGAAATCCCCGGGCTTAACCTGGGAATTGCGCTTGAAACTAGCGGGCTTGAGTGCGGCAGAGGGGGGTGGAATTCCACGTGTAGCAGTGAAATGCGTAGAGATGTGGAGGAACACCGATGGCGAAGGCAGCCCCCTGGGTCGACACTGACGCTCATGCACGAAAGCGTGGGGAGCAAACAGG
Bacteria	Proteobacteria	Gammaproteobacteria	Betaproteobacteriales	Rhodocyclaceae	Denitratisoma	52	0	0	52	0	TACGTAGGGTGCGAGCGTTAATCGGAATTACTGGGCGTAAAGCGTGCGCAGGCGGCGCCATAAGACAGATGTGAAATCCCCGGGCTTAACCTGGGAACTGCGTTTGTGACTGTGGTGCTTGAGTACGGCAGAGGGGGGTGGAATTCCACGTGTAGCAGTGAAATGCGTAGAGATGTGGAGGAACACCGATGGCGAAGGCAGCCCCCTGGGCTAGTACTGACGCTCATGCACGAAAGCGTGGGTAGCAAACAGG
Bacteria	Proteobacteria	Gammaproteobacteria	Enterobacteriales	Enterobacteriaceae	Enterobacteriaceae	850	0	850	0	21	TACGGAGGGTGCAAGCGTTAATCGGAATTACTGGGCGTAAAGCGCACGCAGGCGGTCTGTTAAGTCAGATGTGAAATCCCCGGGCTTAACCTGGGAACTGCATTTGAAACTGGCAGGCTTGAGTCTCGTAGAGGGGGGTAGAATTCCAGGTGTAGCGGTGAAATGCGTAGAGATCTGGAGGAATACCGGTGGCGAAGGCGGCCCCCTGGACGAAGACTGACGCTCAGGTGCGAAAGCGTGGGGAGCAAACAGG
Bacteria	Proteobacteria	Gammaproteobacteria	Enterobacteriales	Enterobacteriaceae	Enterobacteriaceae	43	0	0	43	0	TACGGAGGGTGCAAGCGTTAATCGGAATTACTGGGCGTAAAGCGCACGCAGGCGGTCTGTCAAGTCGGATGTGAAATCCCCGGGCTTAACCTGGGAACTGCATTCGAAACTGGCAGGCTAGAGTCTCGTAGAGGGGGGTAGAATTCCAGGTGTAGCGGTGAAATGCGTAGAGATCTGGAGGAATACCGGTGGCGAAGGCGGCCCCCTGGACGAAGACTGACGCTCAGGTGCGAAAGCGTGGGGAGCAAACAGG
Bacteria	Proteobacteria	Gammaproteobacteria	Enterobacteriales	Enterobacteriaceae	Lonsdalea	15	0	0	15	0	TACGGAGGGTGCAAGCGTTAATCGGAATGACTGGGCGTAAAGCGCACGCAGGCGGTTTGTTAAGTCAGATGTGAAATCCCCGAGCTTAACTTGGGAACTGCATTTGAAACTGGCAAGCTAGAGTCTTGTAGAGGGGGGTAGAATTCCAGGTGTAGCGGTGAAATGCGTAGAGATCTGGAGGAATACCGGTGGCGAAGGCGGCCCCCTGGACAAAGACTGACGCTCAGGTGCGAAAGCGTGGGGAGCAAACAGG
Bacteria	Proteobacteria	Gammaproteobacteria	Enterobacteriales	Enterobacteriaceae	Pantoea	156	0	156	0	0	TACGGAGGGCGCAAGCGTTAATCGGAATTACTGGGCGTAAAGCGCACGCAGGCGGTCTGTTAAGTCAGATGTGAAATCCCCGGGCTTAACCTGGGAACTGCATTTGAAACTGGCAGGCTTGAGTCTCGTAGAGGGGGGTAGAATTCCAGGTGTAGCGGTGAAATGCGTAGAGATCTGGAGGAATACCGGTGGCGAAGGCGGCCCCCTGGACGAAGACTGACGCTCAGGTGCGAAAGCGTGGGGAGCAAACAGG
Bacteria	Proteobacteria	Gammaproteobacteria	Gammaproteobacteria	Gammaproteobacteria	Gammaproteobacteria	194	0	0	194	0	TACAGAGGGTGCAAGCGTTAATCGGAATTACTGGGCGTAAAGCGTGCGCAGACGGTTGCGTAAGTCAGATGTGAAAGCCCCGGGCTCAACCTGGGAATTGCATTTGAGACTGCGTAGCTAGGGTGCGGAAGAGGGAAGCGGAATTTCCGGTGTAGCGGTGAAATGCGTAGATATCTAGAGGAACGCCTGAGGCGAAAGCGGGGCACTGGGCCGATTCTGACGCTGAGACGCGAAAGCGTGGGGAGCAAACAGG
Bacteria	Proteobacteria	Gammaproteobacteria	Gammaproteobacteria_Incertae_Sedis	Unknown_Family	Acidibacter	170	0	170	0	0	TACAGAGGGTGCGAGCGTTAATCGGAATTACTGGGCGTAAAGCGTGCGTAGACGGTTATGTAAGTCGGGTGTGAAAGCCCCGGGCTCAACCTGGGAATTGCATTCGAGACTGCATAGCTAGGGTGCGGAAGAGGGAAGCGGAATTTCCGGTGTAGCGGTGAAATGCGTAGATATCGGAAGGAACATCAGTGGCGAAAGCGGCTTCCTGGTCCAGCACCGACATTCAGGCACGAAAGCGTGGGGAGCAAACAGG
Bacteria	Proteobacteria	Gammaproteobacteria	Halothiobacillales	Halothiobacillaceae	Thiovirga	21	21	0	0	0	TACGTAGGGTGCAAGCGTTAATCGGAATTACTGGGCGTAAAGCGTGCGCAGGCGGTTATGTAAGACAGGCGTGAAATCCCCGGGCTTAACCTGGGAATTGCGCTTGTGACTGCATAGCTAGAGTGTGCAAGAGGATGGGGGAATTCCTGGTGTAGCAGTGAAATGCGTAGAGATCAGGAGGAACATCAATGGCGAAGGCACCTTTCTGGGGCAACACTGACGCTGAGAGACGAAAGCGTGGGGAGCAAACAGG
Bacteria	Proteobacteria	Gammaproteobacteria	Oceanospirillales	Halomonadaceae	Halomonas	19	0	0	19	0	TACGGAGGGTGCGAGCGTTAATCGGAATTACTGGGCGTAAAGCGCGCGTAGGCGGCGTGATAAGCCGGTTGTGAAAGCCCCGGGCTCAACCTGGGAACGGCATCCGGAACTGTCAGGCTAGAGTGCAGGAGAGGAAGGTAGAATTCCCGGTGTAGCGGTGAAATGCGTAGAGATCGGGAGGAATACCAGTGGCGAAGGCGGCCTTCTGGACTGACACTGACGCTGAGGTGCGAAAGCGTGGGTAGCAAACAGG
Bacteria	Proteobacteria	Gammaproteobacteria	Pseudomonadales	Moraxellaceae	Acinetobacter	79	0	0	79	0	TACAGAGGGTGCAAGCGTTAATCGGATTTACTGGGCGTAAAGCGCGCGTAGGTGGCTAAATAAGTCAAATGTGAAATCCCCGAGCTTAACTTGGGAATTGCATTCGATACTGTTTGGCTAGAGTATGGGAGAGGATGGTAGAATTCCAGGTGTAGCGGTGAAATGCGTAGAGATCTGGAGGAATACCGATGGCGAAGGCAGCCATCTGGCCTAATACTGACACTGAGGTGCGAAAGCATGGGGAGCAAACAGG
Bacteria	Proteobacteria	Gammaproteobacteria	SAR86_clade	SAR86_clade	SAR86_clade	226	0	226	0	366	TACGGAAGGTGCAAGCGTTAATCGGAATTACTGGGCGTAAAGCGCGCGTAGGTGGTTTGTTAAGTTGGATGTGAAAGCCCTGGGCTCAACCTAGGAACTGCATCCAAAACTAACTCACTAGAGTACGATAGAGGGAGGTAGAATTCATAGTGTAGCGGTGGAATGCGTAGATATTATGAAGAATACCAGTGGCGAAGGCGGCCTCCTGGATCTGTACTGACACTAAGGTGCGAAAGCGTGGGTAGCGAACAGG
Bacteria	Proteobacteria	Gammaproteobacteria	SAR86_clade	SAR86_clade	SAR86_clade	46	0	46	0	0	TACGGAAGGCGCAAGCGTTAATCGGAATTACTGGGCGTAAAGCGCGCGTAGGTGGTTTGTTAAGTTGGATGTGAAAGCCCTGGGCTCAACCTAGGAACTGCATCCAAAACTAACTCACTAGAGTACGATAGAGGGAGGTAGAATTCATAGTGTAGCGGTGGAATGCGTAGATATTATGAAGAATACCAGTGGCGAAGGCGGCCTCCTGGATCTGTACTGACACTAAGGTGCGAAAGCGTGGGTAGCGAACAGG
Bacteria	Proteobacteria	Gammaproteobacteria	Steroidobacterales	Steroidobacteraceae	Povalibacter	260	0	0	260	0	TACGTAGGGTGCGAGCGTTAATCGGAATTACTGGGCGTAAAGTGTGCGCAGGCGGCGCTGCAAGTCGAGTGTGAAATCCCCAGGCTTAACCTGGGAACTGCATTCGAGACTGCATTGCTAGAGTATGGGAGAGGGAAGTGGAATTTCCGGTGTAGCGGTGAAATGCGTAGATATCGGAAGGAACATCAGTGGCGAAAGCGACTTCCTGGACCAATACTGACGCTCATGTGCGAAAGCGTGGGGAGCAAACAGG
Bacteria	Proteobacteria	Gammaproteobacteria	Steroidobacterales	Steroidobacteraceae	Steroidobacter	240	0	0	240	0	TACAGAGGGTGCGAGCGTTAATCGGAATTACTGGGCGTAAAGCGCGCGTAGGCGGCTTTGCAAGTCGGGTGTGAAATCCCCAGGCTTAACCTGGGAACTGCATTCGAGACTGCATTGCTAGAGTATGGGAGAGGGAAGTGGAATTTCCGGTGTAGCGGTGAAATGCGTAGATATCGGAAGGAACATCAGTGGCGAAAGCGACTTCCTGGACCAATACTGACGCTCATGTGCGAAAGCGTGGGGAGCAAACAGG

Bacteria	Proteobacteria	Gammaproteobacteria	Xanthomonadales	Xanthomonadaceae	Pseudoxanthomonas	28	0	0	28	82	TACGAAGGGTGCAAGCGTTACTCGGAATTACTGGGCGTAAAGCGTGCGTAGGTGGTGGCTTAAGTCCGTTGTGAAAGCCCTGGGCTCAACCTGGGAATTGCAGTGGATACTGGGTCACTAGAGTGTGGTAGAGGGTGGCGGAATTCCCGGTGTAGCAGTGAAATGCGTAGAGATCGGGAGGAACACCCGTGGCGAAGGCGGCCACCTGGGCCAACACTGACACTGAGGCACGAAAGCGTGGGGAGCAAACAGG
Bacteria	Proteobacteria	Proteobacteria	Proteobacteria	Proteobacteria	Proteobacteria	11	0	11	0	0	AACTTAAGGGCTAGTTTTGTGGGCAAAAGCGTAAAGGGTGTGATAGGCCGTTTTAATTAAAAAGGGTTTTATAATTTAAGAAGGTAAATGGAATTTTTTTGTAGCGGTGGAATGTGTAAATAGAAAAAAGAACTTTAGACGTGAAGACTATTTATTTTTGAGATTATAGTGTGATGGCTAAAACACGAGAGTATGGGGAGCAAACAGG
